# Temporal course of cerebrospinal fluid dynamics and amyloid accumulation in the aging rat brain from three to thirty months

**DOI:** 10.1186/2045-8118-9-3

**Published:** 2012-01-23

**Authors:** Catherine Chiu, Miles C Miller, Ilias N Caralopoulos, Michael S Worden, Thomas Brinker, Zachary N Gordon, Conrad E Johanson, Gerald D Silverberg

**Affiliations:** 1 Department of Neurosurgery, Warren Alpert Medical School, Brown University and Aldrich Neurosurgery Research Laboratories, Rhode Island Hospital, Providence, RI, 02903, USA; 2 Brown Institute for Brain Science, MRI Research Facility, Providence, RI, 02912, USA

**Keywords:** Aging, Alzheimer's disease, CSF turnover, Amyloid accumulation

## Abstract

**Background:**

Amyloid accumulation in the brain parenchyma is a hallmark of Alzheimer's disease (AD) and is seen in normal aging. Alterations in cerebrospinal fluid (CSF) dynamics are also associated with normal aging and AD. This study analyzed CSF volume, production and turnover rate in relation to amyloid-beta peptide (Aβ) accumulation in the aging rat brain.

**Methods:**

Aging Fischer 344/Brown-Norway hybrid rats at 3, 12, 20, and 30 months were studied. CSF production was measured by ventriculo-cisternal perfusion with blue dextran in artificial CSF; CSF volume by MRI; and CSF turnover rate by dividing the CSF production rate by the volume of the CSF space. Aβ40 and Aβ42 concentrations in the cortex and hippocampus were measured by ELISA.

**Results:**

There was a significant linear increase in total cranial CSF volume with age: 3-20 months (*p *< 0.01); 3-30 months (*p *< 0.001). CSF production rate increased from 3-12 months (*p *< 0.01) and decreased from 12-30 months (*p *< 0.05). CSF turnover showed an initial increase from 3 months (9.40 day^-1^) to 12 months (11.30 day^-1^) and then a decrease to 20 months (10.23 day^-1^) and 30 months (6.62 day^-1^). Aβ40 and Aβ42 concentrations in brain increased from 3-30 months (*p *< 0.001). Both Aβ42 and Aβ40 concentrations approached a steady state level by 30 months.

**Conclusions:**

In young rats there is no correlation between CSF turnover and Aβ brain concentrations. After 12 months, CSF turnover decreases as brain Aβ continues to accumulate. This decrease in CSF turnover rate may be one of several clearance pathway alterations that influence age-related accumulation of brain amyloid.

## Background

The cerebrospinal fluid (CSF) circulation is critical to maintaining a healthy environment for the brain, and its functional decline with normal aging and the age-related dementias remains of particular interest and concern. For example, there is decreased CSF production and turnover, diminished clearance of proteins, peptides and other potentially toxic metabolites, altered ion and solute transport, and decreased resistance to oxidative stress [1-3]. CSF is secreted mainly by the choroid plexus (CP) as an ultrafiltrate of blood. It circulates through the cerebral ventricles, leptomeninges, and along central nervous system (CNS) surfaces, reentering the bloodstream, in humans, at the arachnoid villi [4]. In rodents there appears to be a significant amount of CSF absorption via the nasal lymphatics [5]. This continual CSF turnover, defined as the rate at which the CSF volume is completely replaced, is considered to play a key role in the clearance of many solutes from the brain [4]. In human and animal models, CSF production by the CP decreases by nearly 50 percent with age [4,6]. The decrease is associated with striking morphological alterations in the CP: flattening of CP epithelial cells, fibrosis, and increased cellular inclusions [3,7]. Total CSF volume has been shown to increase with age, accompanied by brain atrophy [8]. It is not surprising, then, that CSF turnover, calculated by dividing the CSF production rate by the total CSF volume, substantially decreases with age [3,9].

Reduced CSF turnover may have consequences for clearance of many toxic peptides, proteins, and other metabolites. Recently, it was found that albumin (a soluble CSF protein) levels are elevated in elderly sheep associated with decreased CSF production and turnover [10]. Albumin accumulation in the CSF is typical of other soluble proteins in the CSF which, with aging, may increase as brain concentrations increase. By contrast, in cognitively normal aging human subjects amyloid-beta peptide (Aβ) concentrations in the CSF vary inversely with amyloid accumulation in the brain parenchyma, decreasing as brain Aβ accumulates: a consequence of self-assembly into insoluble fibrils and plaques [11-13]. Lower than normal CSF concentration of Aβ42 is a biomarker for Alzheimer's disease (AD) [14] The same inverse relationship between parenchymal and CSF concentrations of Aβ also appears in animal models of aging and AD [15-17]. Although wild-type rats do not form fibrils and plaques as humans do, there is self-assembly of Aβ into oligomeric forms and self-aggregation in the brain parenchyma as medium and large granular formations when Aβ concentrations increase [18,19].

Enlarged cerebral ventricles and decreased CSF turnover are also associated with decreased CSF Aβ concentrations in non-AD patients [20]. In several studies, patients with normal pressure hydrocephalus, a disease characterized by large ventricles and decreased CSF turnover, presented with AD pathology [21]. Other studies have determined that Aβ clearance is reduced in AD subjects, whereas production is unchanged [22], and that CSF Aβ42 concentration is inversely related to ventricular volume and CSF turnover [23]. Since aging is the single most important risk factor for AD genesis [24], and the relationship between CSF dynamics and brain Aβ accumulation has not been analyzed quantitatively, this study was designed to relate CSF production, volume, and turnover to Aβ concentration in the aging Fischer 344/Brown-Norway (F344/BN) hybrid rat brain at 3, 12, 20, and 30 months.

## Methods

### Animals

Male F344/BN hybrid rats aged 3, 12, 20, and 30 months were examined. F344/BN rats were chosen because they are long-lived and available at most ages up to 30 months from the National Institute of Aging colony. Rats used to measure CSF production (n = 24) were **a separate group **from those used to measure both CSF volume (n = 16) and Aβ concentrations (n = 31). The rats were housed in the Central Animal Facility at Rhode Island Hospital and had food and water *ad libitum*. The Institutional Animal Care and Use Committee (IACUC) at Rhode Island Hospital approved all experiments. Rats were euthanized by intra-peritoneal (i.p.) pentobarbital (125 mg/kg) after the acute experiments were completed. Rats used for ELISA measurements were perfused with phosphate-buffered saline (PBS) via left ventricular cannulation prior to removing the brain.

### CSF Volume Measurement

Total cranial and ventricular CSF volumes were measured using a Siemens 3T TIM Trio MR scanner. The scanner was equipped with an AC88 high performance gradient insert and a 4.5 cm custom volume transmit-receive coil. Rats were imaged under continuous 1.8-2.2% isoflurane anesthesia. T1-weighted MPRAGE data were acquired (slice thickness = 0.2 mm; field of view = 6 cm; matrix = 320 mm^3^; TR = 2700 ms; TE = 3.89 ms; inversion time = 900 ms; flip angle = 9 degrees; acquisition time = 12 min 38 s) for a total of 8 acquisitions. All acquisitions were aligned to the original image and averaged using the Analysis of Functional Neuroimages (AFNI) software package [25]. Images were imported as Neuroimaging Informatics Technology Initiative (NIfTI) files into the image analyzer, Slicer 3D [26-28]. Slicer 3D includes a brightness/contrast function, a normal zoom function, and a pixel-by-pixel magnifying glass that allows the user to determine which pixels in the T1-weighted images are gray or black. Fiducial marker points were placed only on black pixels. From these fiducial-marked images, the FastMarching Segmentation module builds a 3D model from surrounding pixels with the same gray scale value [29]. Ventricular CSF spaces (lateral ventricles, third ventricle, cerebral aqueduct, and fourth ventricle) and the subarachnoid space over the brain were marked with fiducial points in each slice, and total cranial CSF volume was calculated from the sum of ventricular CSF and subarachnoid CSF volumes using the FastMarching Segmentation module. Volume analysis was performed by one reader for consistency.

### CSF Production and Turnover Rate Calculation

CSF production was calculated as a function of dye-dilution using ventriculo-cisternal (V-C) perfusion with Blue Dextran 2000 dye (Sigma, St. Louis, MO, USA). Complete methodological details have been previously described [30]. Briefly, the rats were anesthetized with i.p. pentobarbital and immobilized in a stereotaxic frame. Two stainless steel 27-gauge cannulae were inserted, one into each lateral cerebral ventricle. Blue Dextran 2000, at 5 mg/ml, was perfused as mock CSF through the cannulae at 2 μl/min. A third cannula was inserted into the cisterna magna for CSF collection. Intraventricular pressures were monitored by T-connectors to the infusion cannulae. CSF samples were collected every 20 min, and blue Dextran concentration was determined using a spectrophotometer measuring absorbance at 620 nm. The rate of CSF formation was calculated using the following formula: V_f _= V_i _× [(C_i _- C_o_)/C_o_], where V represents the rate of CSF flow, C the concentration of dye, and the subscripts f, i, and o represent formation, inflow, and outflow [30]. CSF turnover was calculated by dividing the mean CSF production (μl/min) by the mean CSF volume (μl) in each age group and multiplying by 1440 min/day, thus obtaining a turnover value in day^-1^.

### Aβ Accumulation

The fronto-parietal cortex plus the complete hippocampal formation was excised from the rat brains post-perfusion with PBS. These samples were then thoroughly ground into a powder over a bed of liquid nitrogen, and 250 mg of that powder was taken for the homogenization and protein extraction. The concentration of Aβ40 and Aβ42 in cortical and hippocampal rat brain samples was measured using a sandwich ELISA method. Details can be found in our previous publication [31]. High-sensitivity ELISA kits (Wako Chemicals, Catalog # 294-64701 and 292-64501, Richmond, VA, USA) were chosen to reduce background and to improve detection of Aβ40 (sensitivity: 0.049 pmol/L) and Aβ42 (sensitivity: 0.024 pmol/L). Both kits employ a two binding site sandwich ELISA system designed to specifically detect Aβ40 and Aβ42. A monoclonal antibody directed against Aβ11- 28 (BNT77) was used as the capture antibody. It is designed to detect both full-length Aβ as well as Aβ with a truncated or modified N-terminus. For specific detection of Aβ40, the monoclonal antibody BA27 directed against the C-terminal portion of Aβ40 was used, and for Aβ42, BC05 directed against the C- terminal portion of Aβ42 was used. Total protein in each sample was determined using a BCA Protein Assay kit (Pierce, Rockford, IL, USA), with absorbance read at 562 nm. Total Aβ concentrations were expressed as pg/mg total protein.

### Statistical Analysis

All statistics were conducted using GraphPad Prism v4.01 (GraphPad Software, San Diego, CA, USA). Data values for CSF volume and CSF production were first subject to analysis of variance (ANOVA) and then to Bonferroni's multiple comparison test. CSF turnover was calculated for each age group from the means of the CSF production values and the CSF volume measurements. Significance was set at *p *< 0.05.

## Results

### CSF Dynamics

**Figure 1:**
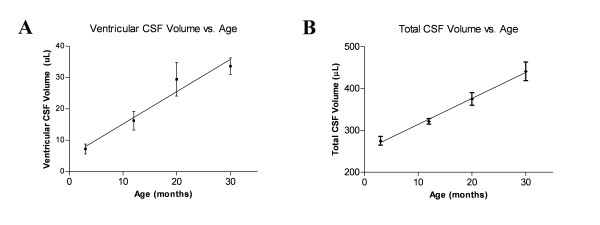
**Age-related increase in CSF volume**. A) Graph of ventricular CSF volume plotted against age (n = 4 for all age groups). Error bars represent SEM. B) Graph of total cranial CSF volume plotted against age (n = 4 for all age groups). Error bars represent SEM. Note that both ventricular and total cranial CSF volumes appear to increase linearly with age.

**Figure 2:**
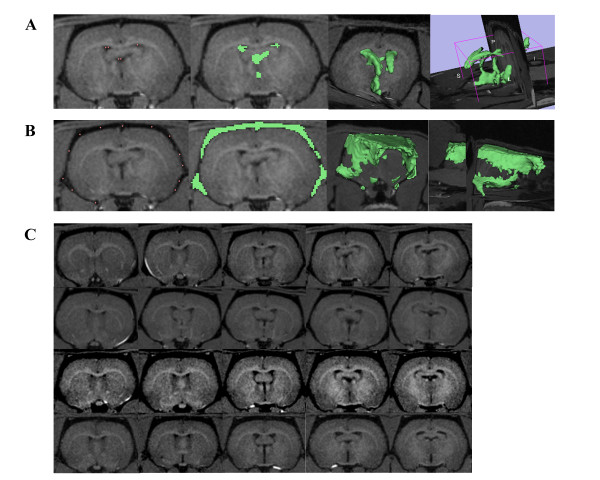
**Total CSF volume**. A) CSF in the ventricles was measured by placing fiducial points (pink dots) on black pixels within the region of interest. FastMarching Segmentation in Slicer 3D generates a rough sketch of the region of interest (green areas), as well as a 3D model. Volume is calculated from this model. B) Similar steps were taken to measure CSF in the subarachnoid space. All measurements were done by one reader for consistency. C) T1-weighted MR images. Each row represents five coronal slices from one rat brain (top row = 3 month, second row = 12 month, third row = 20 month, bottom row = 30 month). Images were magnified and brightened for printing purposes.

Ventricular CSF volume (lateral, third, and fourth ventricles) increased linearly with age, *p *< 0.001, r^2 ^= 0.72 (Figure 1A). The mean ± standard error of the mean (SEM) ventricular CSF volume for rats at 3 months was 7.20 ± 1.6 μL (n = 4), at 12 months it was 16.2 ± 3.0 μL (n = 4), at 20 months it was 29.5 ± 5.4 μL (n = 4), and at 30 months it was 33.6 ± 2.6 μL (n = 4). ANOVA, corrected for multiple comparisons, revealed a significant difference between 3 and 20 months (*p *< 0.01), 3 and 30 months (*p *< 0.001), and 12 and 30 months (*p *< 0.05). Bonferroni's method was used to adjust the level of significance. Figure 2A shows a 3D model of ventricular CSF volume.

Total cranial CSF volume also increased linearly with age, *p *< 0.001, r^2 ^= 0.99 (Figure 1B). The mean ± SEM total cranial CSF volume for rats at 3 months of age was 275 ± 21 μL (n = 4). The 12 month value was 321 ± 13 μL (n = 4). The value at 20 months was 375 ± 30 μL (n = 4), and at 30 months it was 441 ± 45 μL (n = 4). ANOVA, corrected for multiple comparisons, revealed a significant difference between 3 and 20 months (*p *< 0.01), 3 and 30 months (*p *< 0.001), 12 and 30 months (*p *< 0.0001), and 20 and 30 months (*p *< 0.05). Bonferroni's method was used to adjust the level of significance. Figure 2B shows a 3D model of subarachnoid CSF volume, and Figure 2C shows representative T1-weighted MR images for the four age groups taken at several coronal planes.

**Figure 3:**
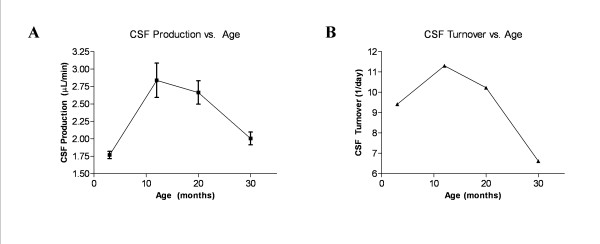
**CSF production and turnover rate as a function of age**. A) Graph of CSF production plotted against age (3 months, n = 6; 12 months, n = 8, 20 months, n = 3; 30 months, n = 7). Error bars represent SEM. B) Graph of CSF turnover plotted against age. CSF turnover is calculated from the means of CSF production and volume measurements. Both production and turnover are non-linear and show an initial increase to 12 months with a subsequent decrease to 30 months. Note the marked decrease in CSF turnover between 20 and 30 months compared to the turnover rate prior to 20 months. The late turnover decrease is due, in large part, to the marked increase in CSF volume, whereas the initial increase is influenced more by the CSF production rate.

CSF production varied significantly with age, *p *< 0.0008 (Figure 3A). The mean ± SEM production rate for rats at 3 months was 1.77 ± 0.05 μL/min (n = 6), at 12 months it was 2.84 ± 0.25 μL/min (n = 8), at 20 months 2.66 ± 0.17 μL/min (n = 3), and at 30 months 2.00 ± 0.09 μL/min (n = 7). Production increased between 3 and 12 months (*p *< 0.01) and decreased between 12 and 30 months (*p *< 0.05). There was no significant difference between 3 and 30 month, 3 and 20 month, and 12 and 20 month production rates (*p *> 0.05), although a continuous decreasing trend is apparent from 12 to 30 months. CSF turnover increased initially between 3 and 12 months (9.41 day^-1 ^to 11.3 day^-1^) and then decreased to 20 months (10.23 day^-1^) and further to 30 months (6.62 day^-1^) (Figure 3B).

### Aβ Accumulation and CSF Turnover Rate

**Figure 4:**
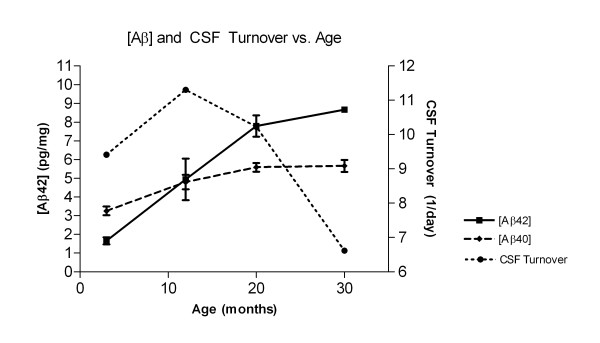
**Graphs of the concentration of Aβ40 and Aβ42 in cerebral cortex plus hippocampus, and CSF turnover plotted against age**. Aβ40 (dashed line, diamonds) and Aβ42 (solid line, squares) in pg/mg total protein concentrations are plotted on the left ordinate (3 months, n = 8; 12 months, n = 8; 20 months, n = 8; 30 months, n = 7). CSF turnover (dotted line, circles) is plotted on the right ordinate. Error bars for Aβ are SEM. CSF turnover is calculated from the means of CSF production and volume measurements. Note that Aβ40 and Aβ42 concentrations increase before there is a significant decrease in CSF turnover. Only later (after 12 months) in the lifespan of the F344/BN rat is there an inverse relationship between Aβ accumulation and the CSF turnover rate.

The concentrations of parenchymal Aβ42 and Aβ40 in specimens of cerebral cortex plus hippocampus increased significantly with age (*p *< 0.001). The mean ± SEM value for Aβ42 in rats at 3 months was 1.65 ± 0.19 pg/mg total protein (tp) (n = 8), at 12 months 4.94 ± 1.11 pg/mg tp (n = 8), at 20 months 7.79 ± 0.57 pg/mg tp (n = 8), and at 30 months 8.67 ± 0.13 pg/mg tp (n = 7). ANOVA with Bonferroni's multiple comparison adjustment revealed a significant difference between 3 and 12 months (*p *< 0.01), 3 and 20 months (*p *< 0.001), 3 and 30 months (*p *< 0.001), 12 and 20 months (*p *< 0.05), and 12 and 30 months (*p *< 0.01). The mean ± SEM value for Aβ40 in 3 months old rats was 3.25 ± 0.23 pg/mg tp (n = 8), at 12 months 4.79 ± 0.39 pg/mg tp (n = 8), at 20 months 5.58 ± 0.23 pg/mg tp (n = 8), and at 30 months 5.66 ± 0.32 pg/mg tp (n = 7). There was a significant difference between 3 and 12 months (*p *< 0.01), 3 and 20 months (*p *< 0.001), and 3 and 30 months (*p *< 0.001). Aβ42 and Aβ40 concentration increased rapidly between 3 and 12 months, followed by a decreased rate of rise in Aβ40 between 12 and 30 months, approaching a steady state between 20 and 30 months. Aβ42 continued to rise to 20 months and then the rate of increase slowed to 30 months. There was a reversal of the Aβ40/Aβ42 ratio in brain parenchyma with advancing age. Figure 4 shows the accumulation of Aβ40 and Aβ42 plotted with the changes in CSF turnover with age.

## Discussion

This study used aging F344/BN rats to temporally relate CSF volume, production and turnover with brain Aβ40 and Aβ42 accumulation. A linear increase in ventricular and total cranial CSF volumes with age and a non-linear relationship between CSF production and age were found. The CSF turnover rate estimates how many times the entire CSF volume is replaced per day and was found to increase from 3 to 12 months and decrease from 12 to 30 months. The significant increase in CSF production from 3 to 12 months and its subsequent decrease to 30 months highly influenced the shape of the turnover curve. There was a rapid increase of cortical and hippocampal Aβ40 and Aβ42 to about 12 months, after which the rate of increase in Aβ40 slowed. Aβ42 continued on the same rising slope to 20 months after which the slope decreased. Both Aβ40 and Aβ42 approached a steady state concentration in the brain by 30 months. The reversal of the Aβ40/Aβ42 ratio in the brain with advancing age was an interesting finding.

The cranial CSF spaces were imaged by MRI. Fiducial points were placed within these spaces to construct a 3D model of the lateral, third, and fourth ventricles, and the subarachnoid space around the brain to calculate total CSF volume. Some measurement error may have been introduced by the observer; however, it would have been consistent throughout the age groups, and our results are within the range of measurements reported by others for ventricular and total cranial CSF volumes [32-35].

Determining the causes of Aβ accumulation in the brain with aging may be crucial to the development of effective AD therapies. Aβ accumulates as a result of an imbalance between production and clearance [22]. Studies suggest that overall brain Aβ accumulation in aging and in AD is not due to an increase in Aβ production, but rather a decrease in Aβ clearance from the brain [22,31,36], although some increase in production cannot be completely excluded by these studies. Several pathways for Aβ clearance exist: i) active transport via receptors at the blood-brain barrier (BBB) and blood-CSF barrier (BCSFB), ii) *in situ *enzymatic degradation and iii) CSF bulk flow and turnover.

Previous reports have shown Aβ clearance pathway alterations with age. There is a significant decrease in Aβ efflux transporter expression with age and an increase in influx transporter expression at the BBB [31,36]. Interestingly, the opposite trend is true for Aβ transporter expression at the BCSFB, perhaps a compensatory response to the overall decrease in BBB Aβ clearance [37]. Studies have also shown decreases in Aβ-degrading enzymes with age [38]. CSF turnover, therefore, may become increasingly more important for metabolite clearance in the aging brain [4].

CSF turnover is a modulator of CSF and brain protein concentrations [9,10]. Although it appears that there is no association between Aβ accumulation in brain parenchyma and CSF turnover rates in the young rats, there may be an inverse relationship between the two in the elderly rats, as part of an age-related multi-factorial decrease in Aβ clearance via the BBB and CSF systems. Clearly, Aβ concentrations in brain increase while CSF production and turnover increase up to 12 months of age. Preston (2001) also found that Aβ begins to accumulate before there is any decrease in CSF production and turnover [3]. However, CSF turnover in association with other age-dependent Aβ clearance defects may contribute to the overall increase in Aβ brain concentrations with advancing age. This temporal association between Aβ and CSF dynamics in the older rats does not prove a cause and effect relationship, but one may argue that the decrease in CSF turnover is a contributing factor. Improving CSF turnover by shunting these rats may show a causal relationship between the two if shunting lowers the rate of Aβ accumulation in aging. Such a study would require measurement of brain, CSF and blood Aβ concentrations in both shunted and sham-operated age-matched controls.

The procedure for measuring CSF production, V-C perfusion, is extremely invasive, and the rats experienced high mortality rates, particularly in the elderly. However, the number of rats at 3 and 12 months was sufficient for showing a significant rise in CSF production, and the decrease from 12 to 30 months fits with existing studies [3,9,10]. In a study by Preston (2001) using Fischer 344 inbred rats, CSF production was shown to increase from 3 to 19 months (1.21 ± 0.27 to 1.48 ± 0.53 μL/min) and then significantly decrease to 30 months (0.65 ± 0.16 μL/min). A 12 month measurement was not included in this study, but judging from our data (see Figure 3A) it would likely have been higher than the 19 month production rate [3]. Because this study and the Preston study used similar rat strains and found similar production results, we believe that the technical difficulties associated with our methods are not substantive.

## Conclusion

There is an increase in CSF production and turnover from 3 to 12 months in this rat aging model at a time when Aβ concentrations increase, showing that before 12 months of age there is no relationship between amyloid deposition in the brain and CSF production and turnover alterations. CSF turnover is inversely related to brain Aβ accumulation in elderly rats. The decreasing CSF turnover rate in older rats suggests a possible therapeutic target to improve CSF solute clearance by shunt insertion in the aging F344/BN rat.

## Abbreviations

Aβ: amyloid-beta peptide; Aβ40: 40 amino acid Aβ; Aβ42: 42 amino acid Aβ; AD: Alzheimer's disease; ANOVA: analysis of variance; BBB: blood-brain barrier; BCSFB: blood-CSF barrier; CP: choroid plexus; CSF: cerebrospinal fluid; ELISA: enzyme-linked immunosorbent assay; F344/BN: Fischer 344/Brown-Norway hybrid rat; IACUC: Institutional animal care and use committee; i.p: intra-peritoneal; MRI: magnetic resonance imaging; AFNI: Analysis of Functional Neuroimages; NIfTI: Neuroimaging Informatics Technology Initiative; 3D: 3-dimensional; PBS: phosphate-buffered saline; SEM: standard error of the mean; tp: total protein; V-C: ventriculo-cisternal.

## Competing interests

There are no actual or potential competing interests (conflicts of interest) to disclose for any of the authors or their families.

## Authors' contributions

CC wrote the first draft of the manuscript, did the MRI volume measurements, and did the CSF turnover calculations. MCM did the ELISA measurements for Aβ40 and Aβ42. INC measured the CSF production rates by V-C perfusion. MW did the MRI scanning and contributed the CSF space software. TB analyzed the data statistically and edited the manuscript. ZNG helped develop the V-C perfusion technique in the lab. CEJ co-directed the research and critically read the manuscript. GDS conceived the research plan, directed the research and edited the manuscript. All authors have read and approved the final version of the manuscript.
